# A Diffusion-Based Data Augmentation Framework for Few-Shot Fault Diagnosis of Intelligent High-Speed Train Components

**DOI:** 10.3390/s26103091

**Published:** 2026-05-13

**Authors:** Jianjun Xu, Qingbin Tong, Ruize Zhu, Shouxin Du, Jilong Zhao, Xuedong Jiang, Baohua Wang

**Affiliations:** 1 School of Electrical Engineering, Beijing Jiaotong University, Beijing 100044, China; jjxu@bjtu.edu.cn (J.X.); 25110530@bjtu.edu.cn (R.Z.); 24110431@bjtu.edu.cn (S.D.); 24121370@bjtu.edu.cn (J.Z.); xdjiang@bjtu.edu.cn (X.J.); bhwang@bjtu.edu.cn (B.W.); 2 Key Laboratory of Vehicular Multi-Energy Drive Systems, Beijing Jiaotong University, Ministry of Education, Beijing 100044, China

**Keywords:** fault diagnosis, imbalanced samples, diffusion model, high-speed train

## Abstract

**Highlights:**

**What are the main findings?**

- MR-DDIM enables class-conditional generation of fault signals under few-shot conditions.
- WT-UNet with FiLM, spectral constraints, and log-σ regularization improves fidelity and distribution alignment of generated samples.

**What are the implications of the main findings?**

- The method alleviates data scarcity and class imbalance in intelligent railway fault diagnosis.
- It provides a potential data augmentation tool for condition monitoring of high-speed train components.

**Abstract:**

Few-shot fault diagnosis of intelligent high-speed train components remains challenging because fault samples are scarce and highly imbalanced. To address this issue, this paper proposes MR-DDIM, a class-conditional diffusion-based data augmentation framework for generating high-fidelity fault vibration signals from limited labeled data. A WT-UNet denoising backbone is developed by combining one-dimensional wavelet convolution with Feature-Wise Linear Modulation (FiLM) to capture multiscale time–frequency structures and enable class-controllable generation. To improve training stability and spectral fidelity, log-σ regularization and a multi-resolution STFT consistency loss are introduced into the optimization process. In addition, this paper proposed the multi-resolution spectral correlation coefficient (MR-SCC) and class-intrinsic maximum mean discrepancy (cMMD) to evaluate generation quality from spectral and distributional perspectives. Experiments on the BJTU-RAO datasets show that the proposed method can generate fault samples with high spectral consistency and reasonable intra-class diversity, thereby improving the robustness of downstream few-shot fault diagnosis. The results indicate that MR-DDIM provides an effective data augmentation solution for intelligent fault diagnosis in high-speed railway systems.

## 1. Introduction

As a typical safety-critical complex system, the health status of key components such as axle box bearings, gearboxes/transmission systems, traction motors, and bogie running gear in intelligent high-speed railways directly affects the safety of train operation and maintenance costs. Under high-speed, heavy load, long-distance, and multi-disturbance conditions, component degradation often presents early weak fault characteristics submerged in strong background noise, significant coupling of operating conditions, and complex fault modes, which make online state monitoring and intelligent diagnosis for actual lines face the contradiction of limited available data and high generalization requirements for a long time [1,2,3]. In recent years, a systematic research framework has been formed for intelligent fault diagnosis of key components of high-speed trains, evolving from sensor layout to signal processing and then to learning models. However, how to maintain stable and reliable diagnostic performance under complex working conditions remains one of the core issues.

The state monitoring data represented by vibration signals contains rich structural dynamics and fault impact information, which is an important basis for diagnosing key components of high-speed railways [4]. With the continuous verification of the advantages of deep learning in feature automatic extraction and end-to-end modeling, methods such as convolutional networks and time–frequency fusion networks have been used for fault recognition of typical components, such as axle box bearings, and have achieved good results [5,6,7,8]. However, comprehensive studies generally point out that the performance of deep models is highly dependent on large-scale annotated data that is sufficient and covers multiple operating conditions, while fault samples in real power lines are often long-tailed, have high collection costs, and are difficult to annotate, explaining the gap between “laboratory effectiveness” and “on-site implementation difficulties”. Therefore, there is often a natural problem of small sample size and class imbalance in fault diagnosis in high-speed railway scenarios: on the one hand, key components are mostly in a healthy state, resulting in a much smaller number of fault samples than normal samples; on the other hand, rare or early faults are difficult to reproduce stably, resulting in very few available samples for each type of fault. In response to this issue, researchers have proposed ideas such as transfer learning, domain adaptation, and metric learning to reduce dependence on the scale of annotated fault data. For example, optimization-based classifiers have been investigated for compound gearbox fault diagnosis with limited data. Time–frequency analysis methods have also been developed to detect weak time-varying fault indicators in complex gearbox systems [9,10,11]. In addition, adaptive signal decomposition methods, such as adaptive dynamic mode decomposition, have been used to extract physically meaningful fault components from nonlinear vibration signals. Recent deep learning studies have further improved bearing fault diagnosis under noisy and variable conditions by combining optimization strategies, decomposition methods, and neural classifiers. But their performance is still limited by insufficient intra-class distribution coverage and distribution shift caused by working condition migration.

To alleviate the training difficulties caused by “small sample/imbalance”, data augmentation is one of the most direct strategies. Traditional methods such as oversampling and mixed enhancement (such as SMOTE and Mixup) are simple to implement but often rely on heuristic rules, making it difficult to systematically characterize the true distribution of complex vibration signals on multiscale time–frequency structures. Compared to others, generative models provide stronger distribution learning capabilities. In the field of mechanical fault diagnosis, GAN has been applied to one-dimensional sensing signal generation and data augmentation, and its promoting effect on downstream classification tasks has been verified [12,13,14]. For example, Fu et al. proposed TRA-ACGAN [15], which combines Transformer with ACGAN for small sample bearing fault data augmentation and classification improvement. Wang et al. proposed FTGAN [16], which models in both time and frequency domains to constrain the time–frequency consistency of generated samples. Jiao et al. proposed AWLT-GAN [17], which embeds an adaptive wavelet transform in the generator and combines it with dual discriminators to balance the imbalanced bearing dataset. However, under small sample and multi-condition conditions, GANs may still face training instability and mode collapse, leading to problems such as seemingly reasonable or unreliable cross-condition generalization.

In recent years, diffusion models and score-based generative methods have become important directions in generative modeling due to their stable training, sufficient pattern coverage, and strong ability to reproduce details. Its typical representative DDPM [18] learns data distribution through “forward gradually adding noise—backward gradually removing noise”, while variants such as DDIM [19] further improve sampling efficiency while maintaining quality. Driven by this, diffusion models have begun to be introduced into fault diagnosis and imbalanced data augmentation, such as the DDPM enhancement framework for rolling bearing imbalanced data, which improves synthetic quality and downstream diagnostic performance by reparameterizing residual structures and diversity losses on imbalanced bearing data [20]. TSDM directly models in the time domain to preserve single/multi-frequency fault features [21], and a two-dimensional time–frequency augmentation scheme based on DDIM + GADF characterization verifies the benefits of class imbalance scenarios on CWRU and actual data [22]. Diffusion enhancement based on hybrid diversity loss, parameter transfer, and frequency-domain/time–frequency consistency constraints provides a new approach for data generation based on diffusion models [23,24].

Although diffusion models have shown promise for fault diagnosis data augmentation and have been validated on equipment such as rolling bearings, several challenges still limit their practical deployment in industrial scenarios. First, existing methods lack mechanism-aware modeling of time–frequency multiscale structures. Most diffusion denoising networks are built upon a generic backbone, without explicitly constraining the multiscale bandpass characteristics or the sideband and modulation components that are critical to fault signal formation. This limitation may result in generated signals that appear morphologically similar in the time domain but fail to preserve stable details in key frequency bands. Second, current training objectives and evaluation criteria remain insufficiently adapted to the signal domain. Existing studies typically rely on standard noise regression losses and assess generation quality mainly through downstream diagnostic accuracy, which cannot fully reflect whether the generated signals simultaneously satisfy spectral consistency and adequate intra-class distribution coverage. Moreover, unlike image generation, fault signal generation lacks well-established domain-specific evaluation criteria, making it difficult to explain generation quality and performance differences through accuracy or visualization alone. Third, the effects of sampling hyperparameters and efficiency-oriented settings have not been systematically investigated for diagnostic applications. Since diffusion sampling inherently involves a trade-off between quality and speed, the common practice of using fixed sampling settings is inadequate for practical deployment. Finally, the robustness of diffusion-based generation under complex noise contamination and load disturbances, which are pervasive in real high-speed railway operating conditions, remains largely unexplored. To address the problem of few-shot fault diagnosis, this paper proposes a class-conditional diffusion-based data augmentation framework. A WT-UNet is developed as the denoising backbone to model the multiscale characteristics and class-dependent variations in one-dimensional vibration signals. Specifically, one-dimensional wavelet convolution is introduced to strengthen cross-band representation learning, while FiLM-based conditional modulation is employed to inject fault-label information into multiscale feature streams for fine-grained controllable generation. During training, a noise-scale-aware objective is combined with a multi-resolution spectral consistency constraint to improve both spectral fidelity and intra-class distribution coverage of generated samples. During inference, skipped-step DDIM sampling is adopted to improve generation efficiency, and classifier-free guidance is used to adjust the strength of class-conditional generation. Experiments under few-shot settings are conducted on the BJTU-RAO datasets. In addition, a joint evaluation criterion, MR-SCC and cMMD, is introduced to assess generation quality from the perspectives of spectral similarity and intra-class distribution alignment. Extensive ablation studies on network architecture and loss design further verify the effectiveness of the proposed framework. In this task, the main difficulty is not only to generate plausible time-domain waveforms, but also to preserve fault-related spectral morphology, intra-class variability, and class-discriminative structures under accelerated DDIM sampling. Therefore, the proposed MR-DDIM is designed around the joint requirements of fast sampling, multiscale spectral preservation, class-controllable generation, and diagnosis-oriented evaluation.

The main contributions of this paper are summarized as follows:

1. A diagnosis-oriented MR-DDIM framework is proposed for few-shot fault vibration signal augmentation. Different from directly applying a generic diffusion generator, the proposed framework is designed for accelerated DDIM sampling while preserving fault-related transient impacts, resonance bands, and modulation-related spectral structures.
2. A WT-UNet denoising backbone is developed to improve the parameterization of the diffusion noise predictor for one-dimensional vibration signals. By embedding fixed wavelet analysis–synthesis priors into the denoising network and combining them with FiLM-based conditional modulation, the model enhances multiscale representation and class-controllable generation under limited fault samples.
3. A spectral-regularized diffusion optimization strategy is introduced. The standard noise-prediction objective is complemented by log−σ uncertainty calibration, and MR-STFT consistency loss is applied to the reconstructed clean estimate, thereby improving spectral fidelity and reducing error accumulation during skipped-step DDIM sampling.
4. A generation-quality evaluation protocol based on MR-SCC and cMMD is established to assess generated fault signals from spectral-structure consistency and class-intrinsic distribution alignment. Together with downstream diagnostic evaluation, this protocol provides a signal-domain assessment of whether generated samples are both realistic and diagnostically useful.

## 2. Materials and Methods

### 2.1. Diffusion Model

Diffusion models progressively inject noise into data in a forward process, transforming samples from the data distribution into an approximately isotropic noise distribution. In the reverse process, a learnable denoising generator—often parameterized by noise prediction or a score function—is trained to iteratively remove noise and recover data samples. During generation, the model starts from a simple prior (e.g., standard Gaussian noise) and applies the reverse diffusion process to map the noise back to the sample space of the target data distribution. To improve generation efficiency, we adopt DDIM sampling. Unlike the Markovian step-by-step sampling in DDPM, DDIM defines a non-Markovian implicit sampling trajectory, enabling “skipped-step” sampling with substantially fewer time steps while maintaining competitive generation quality, thereby reducing inference cost [19].

#### 2.1.1. Forward Process

In the forward diffusion process, Gaussian noise is progressively injected into the original signal. Let $x_{0}$ denote a clean sample, and let T denote the total diffusion horizon. A variance schedule $\{\beta_{t}{\}}_{t=1}^{T}$ is predefined, where $0<\beta_{t}<1$, We define $\alpha_{t}=1-\beta_{t},{\bar{\alpha }}_{t}=\prod_{i=1}^{t}\alpha_{i}$ Then, the one-step forward transition is given by

(1) $$q(x_{t}|x_{0})=N\left(x_{t};\sqrt{{\bar{\alpha }}_{t}}x_{0},\left(1-{\bar{\alpha }}_{t}\right)I\right)$$

which is equivalently written in a reparameterized form as

(2) $$x_{t}=\sqrt{{\bar{\alpha }}_{t}}x_{0}+\sqrt{1-{\bar{\alpha }}_{t}}\epsilon ,\;\epsilon \sim N(0,I)$$

Using the above formula, we can obtain the distribution of data $x_{T}$ after *T* diffusion steps from the original data $x_{0}$ and the added Gaussian noise, completing the diffusion of noise in one step. It should be noted that the Gaussian noise introduced during the forward diffusion process is a mathematical noise mechanism for generating models, rather than a physical assumption that the input data are corrupted only by additive white Gaussian noise.

#### 2.1.2. Reverse Process

In DDPM, the reverse generative process is modeled as a Markov chain, and samples are drawn step-by-step from $p_{\theta }(x_{t-1}|x_{t})$, which typically requires all *T* diffusion steps. To improve sampling efficiency, DDIM constructs a non-Markovian implicit reverse process (while keeping the same forward diffusion), enabling “skipped-step” sampling on a subsequence of timesteps. Let k>s denote a jump from timestep *k* to an earlier timestep *s*. DDIM assumes a Gaussian conditional of the form:

(3) $$q_{\sigma }(x_{s}|x_{k};x_{0})=N\left(\mu_{k\to s}(k;x_{0}),\sigma_{k\to s}^{2}\cdot I\right)$$

To avoid notation ambiguity, we express the mean as a linear combination of $x_{0}$ and $x_{k}$:

(4) $$\mu_{k\to s}(x_{k};x_{0})=ax_{0}+bx_{k},\;x_{s}=ax_{0}+bx_{k}+\sigma_{k\to s}\epsilon ,\;\epsilon \sim N(0,I)$$

From the closed-form forward diffusion,

(5) $$x_{t}=\sqrt{{\bar{\alpha }}_{t}}x_{0}+\sqrt{1-{\bar{\alpha }}_{t}}\epsilon ,\;\epsilon \sim N(0,I)$$

so we have

(6) $$\frac{x_{k}-\sqrt{{\bar{\alpha }}_{k}}x_{0}}{\sqrt{1-{\bar{\alpha }}_{k}}}=\epsilon$$

Matching coefficients yields the commonly used equivalent DDIM form:

(7) $$q_{\sigma }(x_{s}|x_{k},x_{0})=N\left(\sqrt{{\bar{\alpha }}_{s}}x_{0}+\sqrt{1-{\bar{\alpha }}_{s}-\sigma_{k\to s}^{2}}\frac{x_{k}-\sqrt{{\bar{\alpha }}_{k}}x_{0}}{\sqrt{1-{\bar{\alpha }}_{k}}},\sigma_{k\to s}^{2}\cdot I\right)$$

In practice, $x_{0}$ is unknown and is estimated via the predicted noise ${\hat{\epsilon }}_{\theta }(x_{k},k)$:

(8) $${\hat{x}}_{0}=\frac{x_{k}-\sqrt{1-{\bar{\alpha }}_{k}}{\hat{\epsilon }}_{\theta }(x_{k},k)}{\sqrt{{\bar{\alpha }}_{k}}}$$

The DDIM update for the jump k→s is then

(9) $$x_{s}=\sqrt{{\bar{\alpha }}_{s}}{\hat{x}}_{0}+\sqrt{1-{\bar{\alpha }}_{s}-\sigma_{k\to s}^{2}}\epsilon_{\theta }(x_{k},k)+\sigma_{k\to s}z,\;z\sim N(0,I)$$

Here $\sigma_{k\to s}$ controls stochasticity: $\sigma_{k\to s}=0$ yields deterministic sampling, while larger values introduce randomness to improve sample diversity. In DDIM sampling with a timestep jump k→s (k>s), the sampling stochasticity is controlled by the variance term $\sigma_{k\to s}$. A standard parameterization introduces a scalar η∈[0,1] to scale the DDPM-consistent variance term:

(10) $$\sigma_{k\to s}(\eta )=\eta \sqrt{{\tilde{\beta }}_{k\to s}},\;{\tilde{\beta }}_{k\to s}=\frac{1-{\bar{\alpha }}_{s}}{1-{\bar{\alpha }}_{k}}\left(1-\frac{{\bar{\alpha }}_{k}}{{\bar{\alpha }}_{s}}\right)$$

Substituting this into the DDIM update yields:

(11) $$x_{s}=\sqrt{{\bar{\alpha }}_{s}}{\hat{x}}_{0}+\sqrt{1-{\bar{\alpha }}_{s}-\sigma_{k\to s}{\left(\eta \right)}^{2}}\epsilon_{\theta }(x_{k},k)+\sigma_{k\to s}(\eta )z,\;z\sim N(0,I)$$

Here η=0 gives deterministic DDIM, while η>0 injects randomness to increase sample diversity (potentially at the expense of fidelity if set too large). For adjacent steps, the formula reduces to the commonly used form:

(12) $$\sigma_{t}(\eta )=\eta \sqrt{\frac{1-{\bar{\alpha }}_{t-1}}{1-{\bar{\alpha }}_{t}}\beta_{t}}$$

Under this sampling framework, generation quality and efficiency are jointly governed by two sets of hyperparameters. The number of sampling steps controls the sampling speed of the inverse process and the discretization accuracy of the reverse process, and thus affects the fidelity and stability of fine-grained details. The stochasticity parameter η, through $\sigma_{k\to s}$ adjusts the magnitude of random perturbations, influencing sample diversity as well as potential spectral fluctuations. It should be emphasized that the total diffusion horizon *T* defines the training noise schedule, whereas the actual generation efficiency of DDIM is determined by the number of selected reverse timesteps; therefore, the acceleration in this work is achieved by skip-step span sample_step under a fixed *T*, rather than by reducing the total diffusion horizon itself.

### 2.2. Convolution Based on Wavelet Transform (WTCov1d)

Fault vibration signals collected from intelligent high-speed trains are typically multiscale and non-stationary, where transient impulses, harmonics, and modulation sidebands coexist across scales. Under few-shot regimes, purely time-domain convolutions tend to over-smooth high-frequency details, leading to distortions in spectral structures. To inject frequency- and scale-aware priors while keeping the parameterization controllable, we embed the analysis–synthesis mechanism of orthogonal wavelets as a fixed analysis–synthesis operator with perfect reconstruction, up to boundary handling. The module performs only light-weight learnable transformations in the sub-band domain, improving cross-band representation while preserving interpretability and training stability in low-data settings.

Given an input $x\in R^{B\times C\times L}$, where *B*, *C*, and *L* denote the batch size, the number of channels, and the signal length, we construct an analysis–synthesis filter bank $\left(h^{l},h^{h}\right)$ and $\left({\tilde{h}}^{l},{\tilde{h}}^{h}\right)$. For a single-level discrete wavelet transform (DWT) and its inverse (IDWT), the low- and high-frequency sub-band coefficients are defined as

(13) $$\left\{\begin{array}{l} L[n]=\sum_{k}h^{l}[k]x[2n-k]\\ H[n]=\sum_{k}h^{h}[k]x[2n-k] \end{array}\right.$$

where the factor 2 denotes down-sampling by 2. The reconstruction can be written as

(14) $$x[n]=\sum_{k}{\tilde{h}}^{l}[k]\left(↑L[n-k]\right)+\sum_{k}{\tilde{h}}^{h}[k]\left(↑H[n-k]\right)$$

where ↑ denotes up-sampling by 2. Accordingly, we denote the wavelet decomposition and reconstruction as an invertible mapping:

(15) $$\left[L,H]=W(x),\;x=W^{-1}(L,H\right)$$

Based on this formulation, we define a single-layer forward mapping of WTConv1d as

(16) $$y=x_{\mathrm{base}}+W^{-1}\left(T([L,H];\theta )\right),\;[L,H]=W(x)$$

where T([L,H];θ) is a lightweight learnable transform applied to the sub-band pair [L,H], enabling limited structure adjustment and cross-band interaction in the sub-band domain. Here, T([L,H];θ) denotes a small learnable 1D convolutional block applied to the concatenated wavelet sub-bands. Let $U=\left[L,H\right]\;$ be the channel-wise concatenation of the low- and high-frequency sub-bands. Then, T is implemented as a local 1D convolution followed by a nonlinear activation and 1×1 channel mixing. The wavelet analysis–synthesis filters remain fixed, and only the sub-band-domain transform parameters θ are learned. Since the learnable parameters are concentrated in θ, the design tends to be more stable under few-shot training. In parallel, a time-domain residual branch $x_{\mathrm{base}}$, implemented by depthwise residual blocks, is used to preserve local temporal sensitivity and complement the sub-band representation. This branch is an internal part of WTConv1d, and its effect is evaluated together with the complete WTConv1d operator through the structural ablation between the Conv1d backbone and the WTConv1d-based backbone. In practice, W and $W^{-1}$ can be implemented by grouped 1D convolution and transposed convolution, respectively. In this study, Haar wavelets are adopted as the fixed orthogonal analysis–synthesis basis in WTConv1d. The number of wavelet decomposition levels is specified according to the network stage: the initial WTConv1d block uses five decomposition levels, while the downsampling WTConv1d blocks use two decomposition levels. This design is not intended to search for the optimal wavelet family, but to introduce a compact multiscale prior into the DDIM denoising backbone. Haar wavelets are selected because their shortest compact support is suitable for preserving localized impulses and abrupt transitions in gearbox fault vibration signals. Meanwhile, single-level decomposition within each WTConv1d block avoids excessive loss of temporal resolution and reduces boundary-related artifacts, which is important for short vibration segments and few-shot training. The hierarchical multiscale representation is further formed by stacking WTConv1d blocks across the downsampling stages of WT-UNet rather than by using a deep wavelet decomposition within a single block.

## 3. Proposed Method

### 3.1. WT_UNet and FiLM

**Figure 1:**
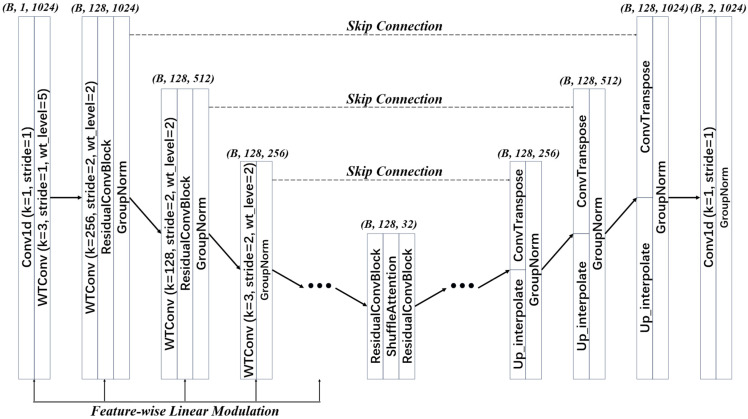
Structure of WT-UNet.

In diffusion-based synthesis of few-shot, non-stationary vibration signals, the noise predictor must simultaneously provide a sufficiently large receptive field and multiscale resolution to capture cross-scale structures (e.g., impulses, modulation, and side-bands), while avoiding spectral-shape degradation during denoising. This requirement becomes more stringent under accelerated sampling (DDIM), where the predictor must perform reliable denoising with fewer timesteps and thus bears a heavier burden per step. Moreover, to ensure controllable class-conditional generation, the noise predictor should exhibit consistent and adjustable responses to both fault labels and diffusion timesteps. To this end, we propose WT-UNet as the denoising backbone for noise prediction. WT-UNet builds upon WTConv1d to explicitly inject scale- and band-aware priors, and employs FiLM-based conditioning to incorporate class labels. These designs enable stable training and physically interpretable multiscale representations with a compact parameterization. The overall architecture of WT-UNet is illustrated in Figure 1.

During down-sampling, WT-UNet applies WTConv1d at each stage and reduces the feature resolution with a stride = 2. Large kernels are used in the first two stages (k = 256 and k = 128) to rapidly enlarge the receptive field and capture long-range structures in non-stationary signals, while deeper stages adopt small kernels (k = 3) to focus on local detail extraction with lower computational cost. By introducing orthogonal wavelet filters as fixed priors and performing lightweight learnable transformations in the sub-band domain, the network enhances receptive-field expansion while preserving signal energy distribution and key-band spectral structures. At the bottleneck, ResBlocks [25] and Shuffle Attention [26] are employed to refine low-resolution features through channel grouping, shuffling, and adaptive reweighting. This helps emphasize fault-sensitive feature subspaces and reduce channel redundancy under few-shot conditions. During up-sampling, two parallel branches are used and fused at the output of each stage. One branch uses learnable transposed convolution to improve feature expressiveness, while the other adopts a smoother geometric up-sampling path to stabilize interpolation. This design reduces checkerboard artifacts and improves alignment with skip-connected features. Finally, WT-UNet uses a dual-head output layer to jointly predict the noise and the variance, making the network better suited to the numerical stability requirements of DDIM sampling.

To achieve unified and controllable modulation over both fault labels and diffusion timesteps, we introduce Feature-Wise Linear Modulation (FiLM) into the multiscale feature streams of WT-UNet for conditional injection. Among them, diffusion steps are an important indicator to guide model training; we embed *t* into a d-dimensional vector, and using sinusoidal positional encoding,

(17) $$e(t)={\left[\sin\left(t\cdot \omega_{k}\right),\cos\left(t\cdot \omega_{k}\right)\right]}_{k=0}^{\frac{d}{2}-1}\in {\mathbb{R}}^{d},\;\omega_{k}=\exp\left(-k\frac{\ln10000}{d/2}\right)$$

**Figure 2:**
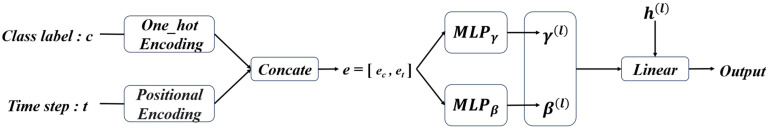
Structure of FiLM.

For the fault label *c*, we map its one-hot encoding to a condition vector *e_c_*. We then concatenate [*e_t_*, *e_c_*] and feed it into a two-layer MLP to produce the per-block (layer *l*) channel-wise scale and bias parameters $\gamma^{\left(l\right)},\beta^{\left(l\right)}\in R^{B\times C}$ (broadcast along the temporal dimension *L*). Given an intermediate feature $h^{\left(l\right)}\in R^{B\times C\times L}$, FiLM modulation is defined as

(18) $$\mathrm{FiLM}\left(h^{\left(l\right)}|e\right)=\mathrm{GN}\left(h^{\left(l\right)}\right)⊙\left(1+\gamma^{\left(l\right)}\right)+\beta^{\left(l\right)}$$

where GN(⋅) denotes Group Normalization and ⊙ is element-wise multiplication (with $\gamma^{\left(l\right)}$, $\beta^{\left(l\right)}$ broadcast over *L*). As shown in Figure 2, this design preserves the temporal resolution and channel layout, while adaptively recalibrating channel responses via learnable scaling and shifting under different timesteps and class conditions. To enhance class-controllable generation during sampling, classifier-free guidance is adopted in the DDIM reverse process. During training, the class condition is randomly replaced with an empty condition with probability *p* = 0.1, enabling the denoising network to learn both conditional and unconditional noise prediction. During sampling, the unconditional prediction and the conditional prediction are linearly combined as follows:

(19) $$\hat{\epsilon }=\epsilon_{u}+\omega \left(\epsilon_{c}-\epsilon_{u}\right),\;\epsilon_{u}=\epsilon_{\theta }(x_{t},t,Ø),\;\epsilon_{c}=\epsilon_{\theta }(x_{t},t,c)$$

where ω is the classifier-free guidance strength. A larger ω enforces stronger class-conditional generation, whereas a smaller ω preserves more diversity by relying more on the unconditional prediction. Here, $\epsilon_{\theta }$ denotes the WT-UNet noise predictor parameterized by θ. It maps the noisy signal $x_{t}$, diffusion timestep *t*, and class condition *c* to the predicted noise with the same shape as $\epsilon_{\theta }:R^{B\times C\times L}\times \{1,\ldots ,T\}\times C\to R^{B\times C\times L}$. The unconditional prediction $\epsilon_{\theta }(x_{t},t,\emptyset )$ is obtained by replacing the class condition with an empty condition.

For reproducibility, the WT-UNet configuration is specified as follows. The input length is 1024 with one channel, and the feature width is fixed to $n_{\mathrm{feat}}=96$. WTConv1d uses the Haar wavelet as the fixed orthogonal basis. The initial WTConv1d block uses a kernel size of 3, a stride of 1, and five decomposition levels, while the downsampling WTConv1d blocks use kernel sizes of 256, 128, and 3 with a stride of 2 and two decomposition levels. Group Normalization with eight groups and SiLU activation is used throughout the convolutional blocks. The bottleneck adopts residual blocks with Shuffle Attention, the decoder uses dual-branch upsampling, and the two-channel output is split into $\epsilon_{\theta }$ and ${\log\sigma }_{\theta }$.

### 3.2. Design of Model Optimization

During training, we follow the diffusion learning framework derived from the evidence lower bound (ELBO) and optimize the denoising network primarily via noise prediction. Beyond noise prediction, to improve numerical robustness under few-shot regimes and skipped-step sampling, we equip the predictor with an additional regularization that outputs a data-dependent log-variance ${\log\sigma }_{\theta }^{2}(x_{t},t,c)$. For a timestep jump, the implicit posterior stochasticity is controlled by η∈[0,1], yielding a target scale $\sigma_{q}$. It should be noted that the exact form depends on the skipping schedule; for adjacent steps, a common form is $\sigma_{q}^{2}=\eta^{2}\frac{1-{\bar{\alpha }}_{t-1}}{1-{\bar{\alpha }}_{t}}\beta_{t}$. We regularize the predicted scale $\sigma_{\theta }$ toward $\sigma_{q}$ via a KL term. We define the log-σ loss as follows:

(20) $$L_{\mathrm{vb}}(\sigma_{\theta }^{2})=E_{x_{0},\epsilon ,t}\left[s_{t}\cdot D_{\mathrm{KL}}\left(N(0,\sigma_{q}^{2})∥N(0,\sigma_{\theta }^{2})\right)\right]$$

For zero-mean Gaussians, the KL reduces to

(21) $$D_{\mathrm{KL}}\left(N(0,\sigma_{q}^{2})∥N(0,\sigma_{\theta }^{2})\right)=\frac{1}{2}\left(\log\frac{\sigma_{\theta }^{2}}{\sigma_{q}^{2}}+\frac{\sigma_{q}^{2}}{\sigma_{\theta }^{2}}-1\right)$$

which discourages the variance head from deviating excessively from the target scale and helps mitigate error accumulation and detail degradation in skipped-step sampling. Moreover, to explicitly enforce spectral-shape fidelity and suppress frequency-domain artifacts, we introduce a differentiable multi-resolution STFT magnitude-consistency loss. Given a real sample $x_{0}\in R^{C\times L}$ and its estimate ${\hat{x}}_{0}$ for a set of STFT configurations $\{(n_{k},h_{k}){\}}_{k=1}^{K}$, we define the following:

(22) $$L_{\text{MR-STFT}}=\frac{1}{K}\sum_{k=1}^{K}{\left\|\left|{\mathrm{STFT}}_{n_{k},h_{k}}(x)\right|-\left|{\mathrm{STFT}}_{n_{k},h_{k}}({\hat{x}}_{0})\right|\right\|}_{1}$$

This loss constrains magnitude spectra without enforcing phase alignment, which improves optimization stability while aligning key-band energy distributions and sideband/modulation structures across multiple time–frequency resolutions. Importantly, we constrain magnitude spectra only, rather than enforcing phase alignment. Phase is highly sensitive to sample-level shifts, window boundaries, and noise perturbations in non-stationary and impulsive signals; directly constraining phase often leads to unstable gradients and degraded optimization. In contrast, magnitude spectra capture the energy distribution and key-band structures that are more relevant to fault mechanisms, providing a stable and diagnosis-oriented frequency-domain regularizer. Moreover, multi-resolution magnitude alignment can be viewed as a soft frequency-band prior. It does not force the model toward rigid templates but encourages the generator to reproduce mechanism-related spectral patterns at multiple scales, thereby reducing over-smoothing and frequency artifacts and improving usability under varying operating conditions.

In implementation, ${\hat{x}}_{0}$ can be reconstructed from the noise predictor at any timestep *t*, allowing MR-STFT to serve as an end-to-end differentiable loss jointly optimized with noise regression.

The final loss function is defined as $L_{\mathrm{total}}=L_{\mathrm{MSE}}+\lambda_{\sigma }L_{\log\sigma }+\lambda_{\mathrm{STFT}}L_{\text{MR-STFT}}$, where $\lambda_{\sigma }$ and $\lambda_{\mathrm{STFT}}$ denote the weights of the log-σ regularization term and the multi-resolution STFT consistency term, respectively. In all experiments, $\lambda_{\sigma }$ and $\lambda_{\mathrm{STFT}}$ were fixed to 0.001 and 0.0005, respectively. The same weighting strategy was used across the ablation and comparison experiments, unless the corresponding loss term was removed.

### 3.3. Design of MR-DDIM

**Figure 3:**
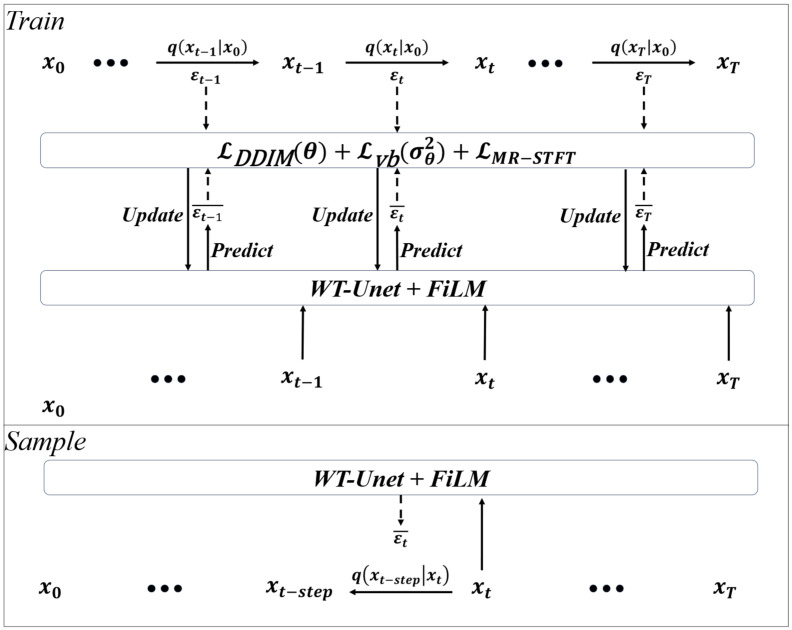
Structure of MR-DDIM.

Based on the previous content, the training and sampling process of the model is illustrated in Figure 3. To reduce computational costs during training, in the forward process of each training round, $x_{t}$ is directly obtained through one-step diffusion from $x_{0}$, and in the subsequent backward process, WT-UNet is input to predict the corresponding noise for parameter optimization. After the model training is completed, ${\hat{x}}_{0}$ is obtained through stride sampling according to requirements.

It should be emphasized that WTConv1d and MR-STFT do not change the basic DDPM/DDIM probabilistic formulation. WTConv1d acts as an architectural inductive bias for the noise predictor $\epsilon_{\theta }(x_{t},t,c)$. Since noise prediction is equivalent to learning the denoising score up to a timestep-dependent scaling factor, the representation structure of the denoising network directly affects the quality of the learned reverse trajectory. The fixed wavelet analysis–synthesis operator introduces a multiscale and band-aware prior, which is suitable for preserving transient impulses, resonance bands, and modulation-related components in vibration signals. MR-STFT loss is applied to the reconstructed clean estimate as (11) and therefore serves as an auxiliary signal-domain regularizer. The standard MSE noise-prediction loss remains the main diffusion objective, while MR-STFT constrains the multi-resolution magnitude spectra of ${\hat{x}}_{0}$. In this sense, MR-STFT provides a spectral-domain regularization of the denoising score learning process, rather than replacing the ELBO-derived diffusion objective.

### 3.4. Generation-Quality Metrics

After completing model training and sampling, we evaluate the model’s quality by comparing the real data and the augmented data. It should be noted that conventional image-generation metrics such as FID [27] are not directly adopted in this study. The original FID relies on Inception features pretrained on natural images, which are not physically aligned with one-dimensional vibration signals. Directly converting vibration signals into two-dimensional images only for FID calculation may introduce additional representation bias and may not reflect fault-related structures such as characteristic spectral peaks, resonance bands, modulation sidebands, and spectral envelopes. To address this, we propose a new evaluation framework that includes multi-resolution spectral correlation coefficient (MR-SCC) and class-intrinsic maximum mean discrepancy (cMMD). Specifically, MR-SCC measures the similarity in time–frequency characteristics between generated and real samples, while cMMD assesses the class-intrinsic distribution discrepancy between generated and real samples. Through these metrics, we can measure the time–frequency consistency of the generated samples while ensuring their alignment in class-intrinsic distribution and their separability between classes.

In this study, MR-SCC and cMMD are computed between generated samples and held-out real fault samples that are not used for training the diffusion model. Thus, these metrics evaluate the alignment between the generated distribution and unseen real data, rather than the similarity between generated samples and the few-shot training samples.

#### 3.4.1. Multi-Resolution Spectral Correlation Coefficient

We apply Short-Time Fourier Transform (STFT) to both real and generated samples to obtain their time–frequency representations $S^{\left(n\right)}(x)$ and $S^{\left(n\right)}(g)$, where *n* represents the STFT configurations. For each pair of generated sample *g* and target sample *x*, we compute their time–frequency correlation at *K* different STFT resolutions $\{(n_{k},h_{k}){\}}_{k}$:

(23) $$S^{\left(n\right)}(x)=\left|{\mathrm{STFT}}_{n,h}(x)\right|\in {\mathbb{R}}^{F\times T},S^{\left(n\right)}(g)=\left|{\mathrm{STFT}}_{n,h}(g)\right|\in {\mathbb{R}}^{F\times T}$$

We define the frequency-domain correlation coefficient $r_{v}$ as

(24) $$r_{v}=\frac{1}{R_{v}}\sum_{r=1}^{R_{v}}\frac{\langle S^{\left(v\right)}(x),S^{\left(v\right)}(g)\rangle }{\left\|S^{\left(v\right)}(x)\right\|\left\|S^{\left(v\right)}(g)\right\|}$$

Here, $R_{v}$ is the sample set, and $\left\langle S^{\left(v\right)}(x),S^{\left(v\right)}(g)\right\rangle$ represents the inner product between the time–frequency features. The range of MR-SCC is [−1, 1], where larger values indicate a higher similarity in the frequency domain between the generated and real samples.

#### 3.4.2. Class-Intrinsic Maximum Mean Discrepancy

Given real sample set $X={\left\{x_{i}\right\}}_{i=1}^{M}\sim P$ and generated sample set $Y={\left\{y_{j}\right\}}_{j=1}^{N}\sim Q$, where *P* and *Q* represent the real and generated data distributions, respectively, we use maximum mean discrepancy (MMD) to measure the class-intrinsic distribution discrepancy between the two. The MMD calculation is as follows:

(25) $${\mathrm{MMD}}^{2}(P,Q)=\frac{1}{M(M-1)}\sum_{i\neq j}\lambda (x_{i},x_{j})+\frac{1}{N(N-1)}\sum_{i\neq j}\lambda (y_{i},y_{j})-\frac{2}{MN}\sum_{i,j}\lambda (x_{i},y_{j})$$

where λ is the similarity function based on a Gaussian kernel. By computing the MMD between generated and real samples, we obtain the class-intrinsic distribution matching score cMMD:

(26) $$c\mathrm{MMD}=\frac{1}{K}\sum_{k=1}^{K}{\mathrm{MMD}}_{k}^{2}$$

To ensure reproducibility, the practical implementation details of MR-SCC and cMMD are summarized in Table 1. Both metrics are computed in a class-wise manner between generated samples and held-out real samples that are not used for diffusion-model training. Therefore, the evaluation measures the alignment between generated samples and unseen real fault samples, rather than the similarity to the few-shot training samples. For MR-SCC, a real-class spectral prototype is first constructed by averaging the log-magnitude spectral envelopes of held-out real samples under each STFT resolution. Each generated sample is then compared with the corresponding class prototype using the Pearson correlation coefficient, and the final MR-SCC is obtained by averaging over STFT resolutions and generated samples. For cMMD, the multi-resolution spectral envelopes are concatenated into a unified feature vector, and the class-wise distribution discrepancy between generated and held-out real samples is measured using an unbiased MMD^2^ estimator with a multi-bandwidth Gaussian RBF kernel.

## 4. Experiment

To validate the effectiveness and robustness of the proposed method, we perform a validation on the real-world high-speed train bogie fault dataset BJTU-RAO, evaluating practicality and generalization under realistic operating conditions. The models provided in this paper were compiled in Python 3.9. The DL framework is PyTorch, running on the RTX 3080 GPU. For reproducibility, the main training and implementation settings are specified as follows. The diffusion step is set to 2000, and the forward process uses a cosine noise schedule. The denoising network is optimized using Adam with a learning rate of 1 × 10^−5^. The number of training epochs is 50K, and the batch size is set to the number of available samples in each fault class. For classifier-free guidance training, the class condition is randomly masked with probability 0.1.

### 4.1. Dataset Introduction

**Figure 4:**
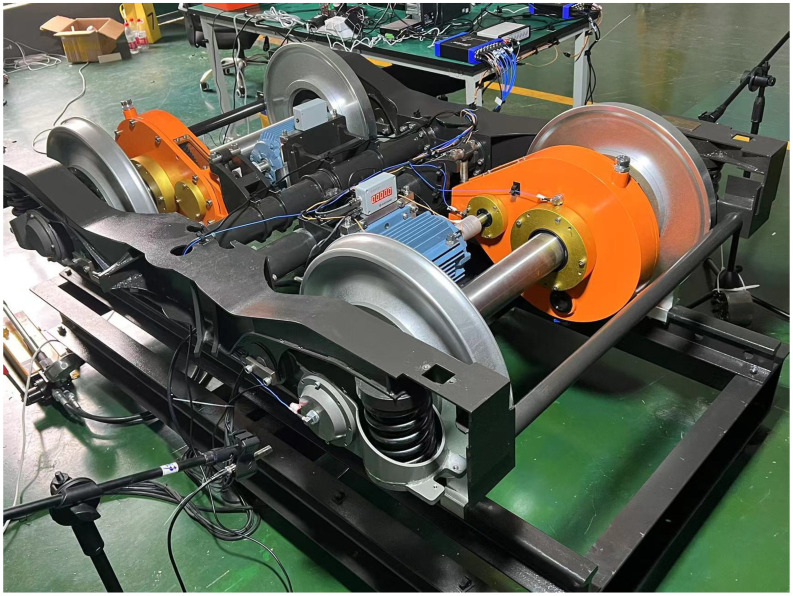
Experimental equipment.

The BJTU-RAO bogie dataset [28] is provided by the State Key Laboratory of Advanced Rail Autonomous Operation, Beijing Jiaotong University. As shown in Figure 4, the experimental platform is built based on real train bogies, and the sensor layout follows actual vehicle measurement points, enabling synchronous acquisition of multi-channel vibration, current, and rotational speed signals from key components such as axle boxes, gearboxes, and traction motors.

In this study, gearbox fault data under the zero-load and 20 Hz operating conditions were selected to construct the experimental dataset. Eight gearbox fault categories were included, covering representative failure types such as root cracks, tooth surface wear, missing teeth, and broken teeth. These faults are commonly encountered in practical bogie transmission systems and exhibit diverse fault characteristics, which makes the generation task challenging. To evaluate the proposed method under different levels of data scarcity, multiple few-shot experimental settings were designed. The detailed training and testing splits are listed in Table 2. Both the training and testing sets consist of real fault samples, and no overlap exists between them.

### 4.2. Training and Generation

During model training, the number of training epochs in each experiment was set to 50K, the batch size was set to the total number of samples in each fault category, and the total number of diffusion steps was fixed at 2000. After training, the diffusion model was used to generate a large-scale augmented dataset for all fault categories. To evaluate the similarity between generated and real samples at the feature level, 400 samples were randomly selected from the augmented dataset for batch-wise comparison with real data. Specifically, the average logarithmic amplitude spectra and their fluctuation ranges were compared between real and generated samples within each class, so as to assess whether the generated fault signals approximate the true data distribution in a frequency-domain statistical sense.

**Figure 5:**
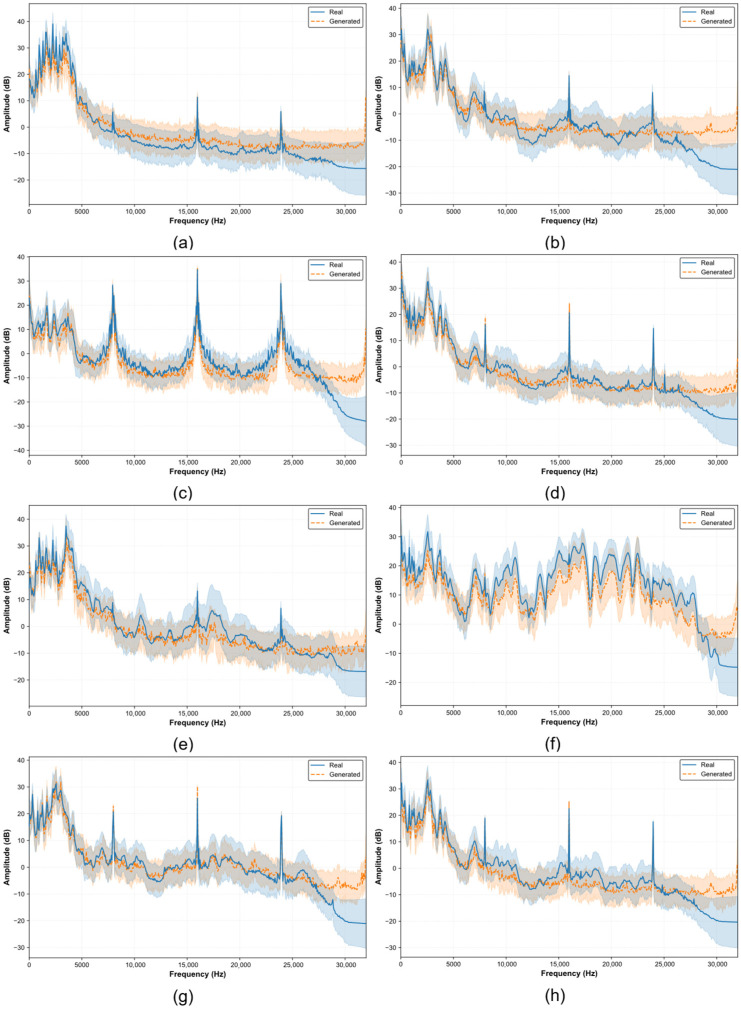
Macro mean and 95% confidence interval between real and generated. (**a**) G1; (**b**) G2; (**c**) G3; (**d**) G4; (**e**) G5; (**f**) G6; (**g**) G7; (**h**) G8.

As shown in Figure 5, the generated and real data exhibit highly consistent peak locations at several prominent spectral peaks, and their shaded fluctuation bands also overlap considerably. In the mid- to low-frequency range, the generated spectra follow the same overall trend as the real spectra, showing a gradual decay with increasing frequency and maintaining a consistent main energy distribution range. This indicates that the proposed model can effectively capture the frequency-band energy distribution of real fault signals. In the high-frequency range, however, noticeable discrepancies can be observed. This is mainly because the spectral amplitude of the real signals rapidly decays toward the noise floor due to the bandwidth limitations of the sensor and sampling system, while the generated signals are not explicitly constrained by strong physical priors in this region. Nevertheless, the energy proportion in this band is extremely low and contributes little to the dominant fault characteristics, so its deviation has limited influence on the overall diagnostic task. It can also be observed that relatively large discrepancies appear in the 10–20 kHz band for some classes, especially in subplots (a), (b), and (h). This phenomenon can be explained from both signal and modeling perspectives. From the signal perspective, the dominant fault-related energy of the selected vibration segments is mainly concentrated in the low- and mid-frequency regions, where characteristic spectral peaks, resonance responses, and modulation-related structures are more evident. In contrast, the 10–20 kHz band is closer to a weak high-frequency region, where the spectral amplitude of real signals rapidly approaches the noise floor and is more sensitive to sensor bandwidth, random local impacts, and measurement noise.

In addition, the widths of the shaded bands reflect the natural variation among different samples at each frequency point. The generated data show fluctuation bands comparable to those of the real data in most frequency ranges, with substantial overlap, indicating that the model is able not only to reproduce the correct average spectral shape, but also to preserve a reasonable degree of intra-class variability. Overall, the proposed method effectively reconstructs the main frequency characteristics, global spectral envelope, and intra-class fluctuation patterns of real signals in the frequency domain. These results suggest that the model captures the characteristic frequencies and harmonic structures of each fault category, rather than merely generating a smooth noise-like spectrum.

**Figure 6:**
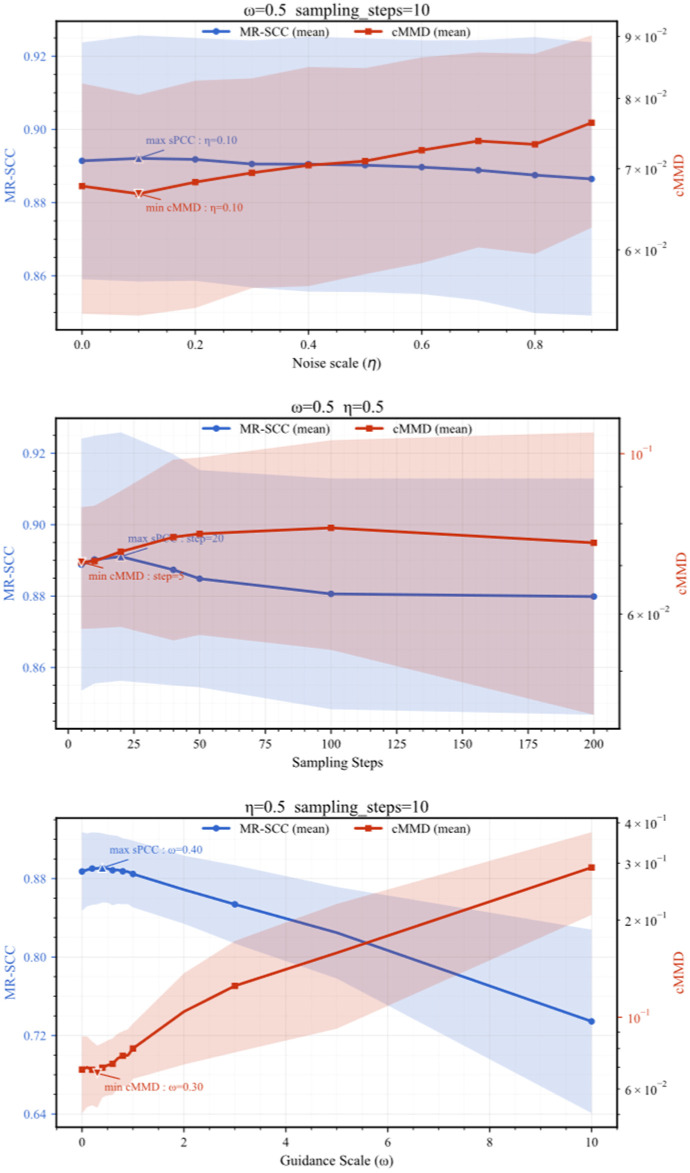
Comparison of different hyperparameters.

At the same time, we also investigated the generation quality under *w*, sample_step, and *η*, and plotted the relationship between the macro mean values of MR-SCC and cMMD and their 95% confidence intervals (CIs). As shown in Figure 6, the solid line represents the macro average of eight classes, and the shadow represents the 95% confidence interval (across classes). It should be emphasized that the real samples used for generation-quality evaluation are from the held-out test set and are not seen by the diffusion model during training. For each hyperparameter setting, MR-SCC and cMMD are first computed within each fault class and then summarized by the macro average over all classes. The shaded region in Figure 6 denotes the 95% confidence interval across classes, calculated as $\bar{x}\pm 1.96s/\sqrt{C}$, where C is the number of fault classes, and s is the standard deviation of the class-wise scores. This reporting protocol reflects the class-level variability of generation quality under different sampling settings. Therefore, the reported MR-SCC, cMMD, and spectral comparisons measure the consistency between generated samples and independent real fault samples, rather than the similarity between generated samples and the few-shot training samples. We can observe that there exists a tolerant optimal band for the guidance coefficient w. Under moderate intensity guidance coefficients, the generated data can achieve both high MR-SCC and low cMMD simultaneously. For the skip-step span sample_step, there is a clear stable generation interval (2–40), beyond which the computational cost of sampling and the quality of data generation will be affected. For the sampling noise ratio *η*, the variation in generation quality with *η* is not significant. Based on experimental results, we can choose *η* ≈ 0.1 in actual operation, which can generate data with both fidelity and diversity. Three sets of ablation (*w*, sample_step, and *η*) indicate that guidance intensity, skip-step span, and randomness control the generation quality from three dimensions: fidelity details, convergence adequacy, and distribution coverage, respectively. The impact of the three on MR-SCC (spectral similarity) and cMMD (intra-class distribution matching) is predictable and controllable.

From the perspective of fault vibration signal generation, these three sampling-related hyperparameters have clear signal-mechanism implications. The guidance strength w controls the degree to which the reverse denoising trajectory is biased toward class-conditional fault patterns. For gearbox fault signals, moderate guidance helps enhance class-discriminative structures, including characteristic spectral peaks, resonance bands, and modulation sidebands. However, an excessively large w may over-sharpen these class-related components and reduce natural intra-class variability, which explains the possible increase in cMMD under strong guidance. The sampling step span determines the discretization accuracy of the DDIM reverse trajectory. Fault vibration signals are typically composed of short-duration impulses, resonance responses, weak high-frequency details, and modulation-related sidebands. When the step span is too large, the reverse process becomes overly coarse, and the model may fail to accurately recover these fine-grained structures, leading to degraded spectral consistency. Therefore, the stable interval of sample_step reflects a balance between sampling efficiency and the preservation of physically meaningful fault-related structures. The stochasticity parameter *η* controls the random perturbation introduced during DDIM sampling. A small nonzero *η* is beneficial for maintaining natural intra-class diversity, such as phase shifts, amplitude fluctuations, and variations in the background noise floor. Nevertheless, excessive stochasticity may introduce non-physical spectral fluctuations and weaken the stability of characteristic frequency components. This explains why *η* has a relatively tolerant range but should not be set too large in practical fault signal generation.

Moreover, if the model merely reproduced near-duplicates of the few-shot training samples, the generated distribution would be expected to collapse around a small number of training instances. In that case, it would be difficult to simultaneously obtain high MR-SCC and low cMMD with respect to held-out real fault samples.

To verify the independent contributions of each design, we conducted ablation experiments in BJTU-RAO small sample experiments, with experimental settings shown in Table 3. Group A examines the UNet backbone and conditional injection, using the B3 training method to replace the pure Conv1d UNet baseline (A0) with WTConv1d (A1) and enable FiLM conditional modulation (A2), ultimately obtaining the backbone WT-UNet + FiLM (A3) in this paper. Group B investigates spectral/physical consistency and sampling control, gradually adding log-σ regularization (B1) and MR-STFT (B2) to the basic noise MSE loss (B0) using the A3 structure. All experiments have the same training epochs, with hyperparameters set to w = 0.5, time step = 20, and eta = 0.5 during sampling. After sampling, pair and evaluate 400 generated samples and 400 real samples. Multiple experiments were conducted to calculate the macro mean of MR-SCC and cMMD. The results are shown in the table.

From the results of structural ablation, the MR-SCC of baseline model A0 is only 0.016, and the cMMD is as high as 0.246, indicating that using ordinary convolution alone is difficult to effectively characterize the multiscale time–frequency characteristics in small sample fault signals during step sampling. After introducing WT Conv, the MR-SCC of A1 increased to 0.734 and the cMMD decreased to 0.122, indicating that wavelet convolution can significantly enhance the model’s ability to characterize cross-band fault modes, improve the spectral consistency and distribution fit of generated samples. In contrast, although introducing only FiLM in A2 increased MR-SCC to 0.759, cMMD only decreased to 0.167, indicating that the conditional modulation mechanism helps enhance class-related feature expression, but still has limited constraints on intra-class distribution. When WT Conv is used in combination with FiLM, A3 achieves the best results in both indicators (MR-SCC = 0.922, cMMD = 0.017), indicating significant complementarity between the two: the former enhances multiscale time–frequency modeling, while the latter improves category conditional modulation capability. After the synergistic effect, it can simultaneously improve spectral fidelity and intra-class distribution consistency. It is worth noting that A2 has a higher number of parameters than A1, but its cMMD performance is actually poor, indicating that the performance improvement does not come from an increase in parameter size, but mainly depends on the degree of matching between module design and task characteristics.

From the ablation results of the loss function, it can be seen that under the basic MSE loss, B0 has achieved an MR-SCC of 0.836 and a cMMD of 0.122, indicating that point-to-point reconstruction constraints can provide some generation ability, but the constraints on spectral details and distribution consistency are still insufficient. After introducing log-σ regularization alone, the MR-SCC of B1 decreased to 0.259, and cMMD increased to 0.156, indicating a significant degradation in performance. This suggests that in the absence of effective spectral constraints, uncertainty weighting may weaken the model’s ability to fit key time–frequency details. In contrast, after introducing MR-STFT loss, the MR-SCC of B2 increased to 0.844, and the cMMD decreased to 0.112, indicating that multi-resolution spectral consistency constraints can effectively improve the fidelity of the time–frequency structure of the generated signal. After further combining with log-σ regularization, B3 achieved the optimal result (MR-SCC = 0.922, cMMD = 0.017), indicating that log-σ regularization can only play a positive role under clear spectral constraints and effectively complements MR-STFT. The degradation of B1 and the improvement of B3 indicate that log-σ regularization should be understood as an uncertainty-calibration term rather than a direct spectral fidelity constraint. When used alone, log-σ may allow the model to treat difficult-to-reconstruct fault-related components, such as transient impacts and weak modulation sidebands, as uncertainty, thereby weakening the reconstruction of key spectral structures. In contrast, MR-STFT loss provides a multi-resolution spectral anchor by constraining the magnitude-spectrum distribution of the reconstructed signal. Under this spectral anchor, log-σ regularization calibrates residual uncertainty around physically meaningful frequency components instead of relaxing their reconstruction. This complementary effect stabilizes skipped-step DDIM sampling and explains why the combination of log-σ and MR-STFT achieves the best MR-SCC and cMMD.

The degradation of B1 and the improvement of B3 indicate that log-σ regularization should be understood as an uncertainty-calibration term rather than a standalone spectral fidelity constraint. When used alone, log-σ regularization does not explicitly indicate which time–frequency structures should be preserved. Therefore, difficult-to-reconstruct fault-related components, such as transient impacts, weak modulation sidebands, and high-frequency resonance details, may be partially absorbed into the uncertainty term, weakening the reconstruction of key spectral structures and leading to degraded MR-SCC and cMMD. In contrast, MR-STFT loss provides a multi-resolution spectral anchor by constraining the magnitude-spectrum distribution of the reconstructed signal. Under this spectral anchor, log-σ regularization mainly calibrates the residual uncertainty around physically meaningful spectral structures instead of relaxing their reconstruction. This complementary effect stabilizes skipped-step DDIM sampling and explains why the combination of log-σ and MR-STFT achieves the best results in B3.

**Figure 7:**
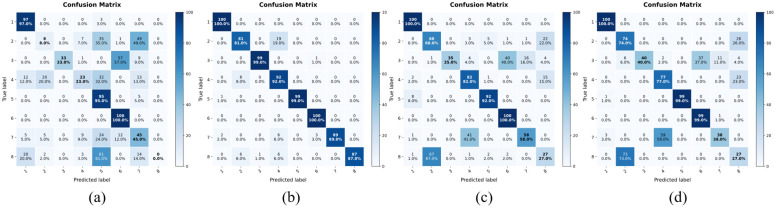
Comparison of confusion matrices for classification tasks: (**a**) No Augment, (**b**) Proposed, (**c**) IResUnet-DM, and (**d**) TSDM.

This study also compares the proposed method with several existing diffusion-based models. Since the purpose of this comparison is to evaluate whether the proposed WT-UNet and spectral regularization improve diffusion-based vibration signal generation under DDIM sampling, we chose two strong and correlated diffusion baselines instead of the independent image domain DDIM model. After training the competing methods under their respective settings, augmented data were generated using both DDPM sampling and DDIM sampling. For fair comparison, the total number of diffusion steps and the DDIM sampling interval were kept consistent with those used in the proposed method. The quantitative results are reported in Table 4. In addition, downstream classification experiments were conducted on the augmented datasets, and the results are shown in Figure 7 and Table 5. The model used for classification tasks is AlexNet [29]. To evaluate the downstream diagnostic usefulness of the generated samples, AlexNet was used as a fixed classifier for all augmentation strategies. The purpose of this experiment is not to optimize the classifier architecture, but to use a relatively lightweight and non-specialized diagnostic model as a probe to assess the quality of the generated data. If the classifier trained with augmented data achieves better performance than the classifier trained only with few-shot real data, the improvement can be attributed to the additional class-discriminative information provided by the generated samples. The training set used in (a) is an unexpanded small sample dataset, (b) is the dataset amplified by the method proposed in this paper, and (c) and (d) are the amplified datasets obtained by IResUnet-DM and TSDM under DDPM sampling, respectively.

The experimental results indicate that, for conventional DDPM-based models, DDIM sampling offers an inherent advantage in sampling efficiency. However, most existing noise prediction networks are originally optimized for the DDPM sampling process. As a result, directly replacing DDPM with DDIM for accelerated inference often leads to a noticeable degradation in generation quality. In contrast, compared with the competing methods, the proposed approach achieves a substantial improvement in generation speed while still effectively preserving the quality and downstream usability of the generated data.

### 4.3. Additional Working-Condition Validation on BJTU-RAO

**Figure 8:**
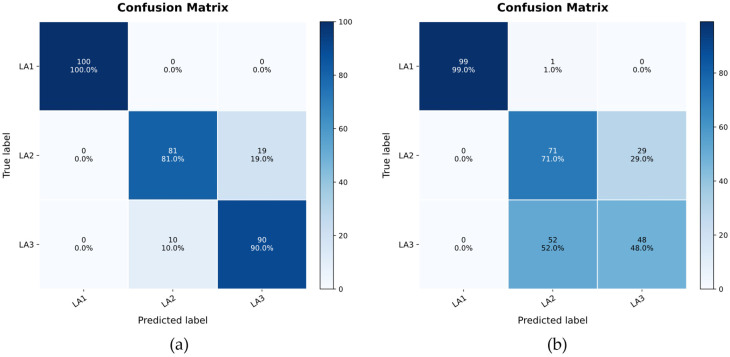
Comparison of confusion matrices. (**a**) After Augment and (**b**) Before Augment.

To further examine whether the proposed MR-DDIM framework is effective beyond the original fixed operating condition, we added additional validation on the BJTU-RAO dataset under additional load–fault-state combinations. In addition to the original 20 Hz and 0 kN setting, two shifted-load conditions at the same rotational speed were considered, namely 20 Hz with −10 kN and 20 Hz with +10 kN. The faulty component is the left axle box. The evaluated data files include M0_G0_LA1_RA0_20Hz_0kN, M0_G0_LA2_RA0_20Hz_−10kN, and M0_G0_LA3_RA0_20Hz_+10kN. For each condition, the same few-shot generation and downstream diagnostic protocol was adopted. All training configurations are consistent with the method in Section 4.2, and the hyperparameters for inference are set to w = 0.5, time step = 20, eta = 0.5. The diffusion model was trained using only limited real samples, while the remaining real samples were held out for generation-quality evaluation and downstream classification testing. Therefore, the reported MR-SCC and cMMD by category are computed using real samples that were not seen by the diffusion model during training. The results in Table 6 show that MR-DDIM maintains reasonable spectral consistency and class-intrinsic distribution alignment under the additional load–fault-state combinations. Compared with the unaugmented few-shot baseline, MR-DDIM improves downstream diagnostic performance and preserves reasonable spectral consistency and class-intrinsic distribution alignment. Meanwhile, the quantitative results are reported in Table 6, and Figure 8 presents the confusion matrix of downstream classification tasks before and after data augmentation. These results indicate that the proposed framework is not limited to the original 20 Hz and 0 kN setting, but can also provide useful augmentation under load-shift conditions within the BJTU-RAO platform.

## 5. Conclusions

In this paper, a class-conditional diffusion-based data augmentation framework, termed MR-DDIM, was proposed for few-shot fault diagnosis of intelligent high-speed train components. To address the challenges of limited fault samples, class imbalance, and insufficient preservation of time–frequency structures in generated vibration signals, a WT-UNet denoising backbone was developed by integrating wavelet-based multiscale priors with FiLM-based conditional modulation. Meanwhile, a joint optimization strategy combining log-σ regularization and multi-resolution STFT consistency loss was introduced to improve training stability, spectral fidelity, and intra-class distribution consistency under fast sampling.

Experimental results on the BJTU-RAO datasets demonstrated that the proposed method can generate fault signals with high spectral similarity and reasonable intra-class diversity under few-shot settings. The generated samples preserved the dominant spectral peaks, global spectral envelope, and fluctuation characteristics of real signals. Ablation studies further verified the complementarity of WTConv and FiLM, as well as the effectiveness of combining MR-STFT with log-σ loss under explicit spectral constraints. In addition, comparative experiments with existing diffusion-based methods showed that the proposed approach achieves a better balance between generation quality and sampling efficiency under DDIM sampling, while also improving the usability of augmented data for downstream fault classification.

Overall, the proposed MR-DDIM framework provides an effective and practical solution for data augmentation in few-shot fault diagnosis of intelligent high-speed railway systems. Future work will further investigate cross-speed, cross-device, and long-term onboard validation under different train platforms, sensor layouts, component types, and real operating disturbances. In addition, robustness under colored noise, impulsive interference, and complex load variations will be systematically evaluated, and adaptive wavelet selection or condition-aware generation strategies will be explored to improve the generalization capability of diffusion-based fault signal augmentation.

## Figures and Tables

**Table 1:** Implementation details of MR-SCC and cMMD.

Item	Setting
Evaluation data	Generated samples vs. held-out real samples
Signal length	1024
STFT window	Hann window
STFT configurations	(nperseg, hop) = (256, 64), (512, 128), (1024, 256)
Spectral representation	STFT magnitude spectrum
Spectral preprocessing	Time-averaged magnitude spectrum, log(1 + |STFT(·)|), frequency-wise z-score normalization
cMMD feature space	Concatenated multi-resolution spectral-envelope features
cMMD kernel	Multi-bandwidth Gaussian RBF kernel
Bandwidth selection	Median heuristic with scales {0.5, 1.0, 2.0, 4.0}
cMMD estimator	Unbiased MMD^2^ estimator

**Table 2:** Dataset splitting.

	No.	Fault Type							
		G1	G2	G3	G4	G5	G6	G7	G8
Train	1	10	10	10	10	10	10	10	10
	2	5	5	5	5	5	5	5	5
	3	2	2	2	2	2	2	2	2
Test	4	400	400	400	400	400	400	400	400

**Table 3:** Ablation experiment.

No.	Setting	Parameters	MR-SCC	cMMD
A0	Baseline-Conv	365.474K	0.016	0.246
A1	A0 + WT_Conv	412.994K	0.734	0.122
A2	A0 + FiLM	513.506K	0.759	0.167
**A3 (Ours)**	**A0 + WT_Conv + FiLM**	**561.026K**	**0.922**	**0.017**
B0	Base MSE Loss	/	0.836	0.122
B1	B0 + log-σ	/	0.259	0.156
B2	B0 + MR-STFT	/	0.844	0.112
**B3 (Ours)**	**B0 +** log-σ **+ MR-STFT**	**/**	**0.922**	**0.017**

**Table 4:** Comparison of MR-SCC and cMMD values among different models.

Model	Sampling Mode	MR-SCC	cMMD
IResUnet-DM	DDPM	0.829	0.116
	DDIM	0.623	0.209
TSDM	DDPM	0.852	0.106
	DDIM	0.619	0.193
**MR-DDIM (Proposed)**	DDIM	**0.922**	**0.017**

**Table 5:** Downstream diagnostic performance using AlexNet.

Method	Accuracy	Macro-Precision	Macro-Recall	Macro-F1
No Augment	0.5012	0.4736	0.5012	0.4290
IResUnet-DM	0.7025	0.7261	0.7025	0.6844
TSDM	0.6925	0.7286	0.6925	0.6781
**MR-DDIM**	0.9337	0.9404	0.9338	0.9348

**Table 6:** Cross load experiment on BJTU-RAO.

Fault Type	Condition	MR-SCC	cMMD
LA1	20Hz_0kN	0.9069	0.0404
LA2	20Hz_–10kN	0.9168	0.0484
LA3	20Hz_+10kN	0.9177	0.0508

## Data Availability

Data are contained within the article.
